# Noninvasive right ventricular work in patients with atrial septal defects: a proof-of-concept study

**DOI:** 10.1186/s12947-023-00306-8

**Published:** 2023-05-20

**Authors:** Jian Wu, Xinyi Huang, Weibin Chen, Yiruo Tang, Xu Chen, Xinyu Wang, Bo Jing, Yuanyuan Sun, Kunhui Huang, Qiumei Gao, Xueming Liu, Maolong Su

**Affiliations:** 1 Department of Ultrasonography, Xiamen Cardiovascular Hospital of Xiamen University, School of Medicine, Xiamen University, Xiamen, China; 2 School of Medicine, Xiamen University, Xiamen, China; 3 Department of Cardiology, Xiamen Cardiovascular Hospital of Xiamen University, School of Medicine, Xiamen University, Xiamen, China

**Keywords:** Echocardiography, Right ventricular myocardial work, Atrial septal defect

## Abstract

**Background:**

Noninvasive right ventricular (RV) myocardial work (RVMW) determined by echocardiography is a novel indicator used to estimate RV systolic function. To date, the feasibility of using RVMW has not been verified in assessing RV function in patients with atrial septal defect (ASD).

**Methods:**

Noninvasive RVMW was analysed in 29 ASD patients (median age, 49 years; 21% male) and 29 age- and sex-matched individuals without cardiovascular disease. The ASD patients underwent echocardiography and right heart catheterization (RHC) within 24 h.

**Results:**

The RV global work index (RVGWI), RV global constructive work (RVGCW), and RV global wasted work (RVGWW) were significantly higher in the ASD patients than in the controls, while there was no significant difference in RV global work efficiency (RVGWE). RV global longitudinal strain (RV GLS), RVGWI, RVGCW, and RVGWW demonstrated significant correlations with RHC-derived stroke volume (SV) and SV index. The RVGWI (area under receiver operating characteristic curve [AUC] = 0.895), RVGCW (AUC = 0.922), and RVGWW (AUC = 0.870) could be considered good predictors of ASD and were superior to RV GLS (AUC = 0.656).

**Conclusion:**

The RVGWI, RVGCW, and RVGWW could be used to assess RV systolic function and are correlated with RHC-derived SV and SV index in patients with ASD.

**Graphical Abstract:**

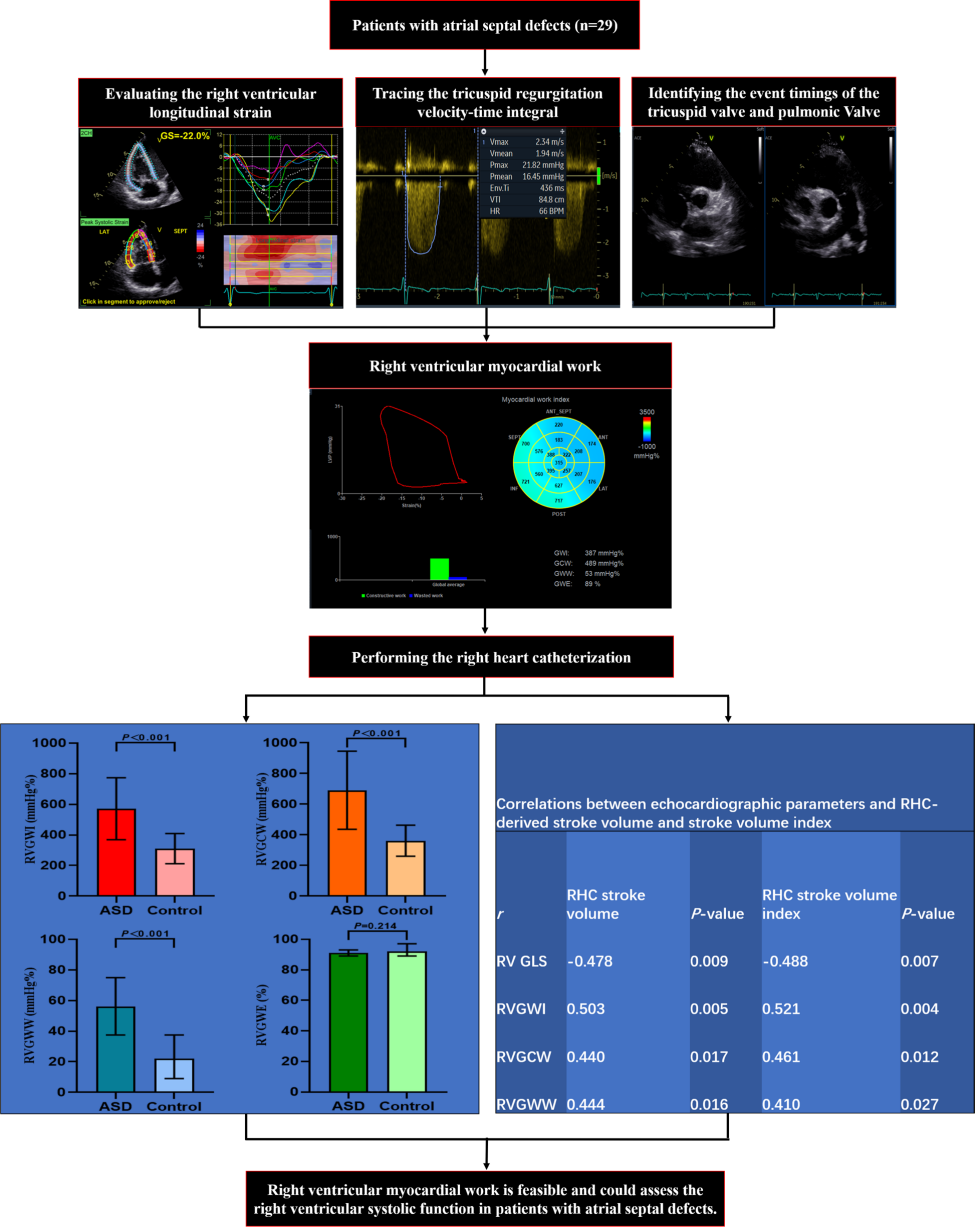

**Supplementary Information:**

The online version contains supplementary material available at 10.1186/s12947-023-00306-8

## Background

Atrial septal defect (ASD) is a common form of congenital heart disease with an estimated prevalence of 1 in 1000 live births [1, 2]. The rate of right ventricular (RV) dysfunction increases in patients with untreated ASD.

Echocardiography plays a crucial role in the evaluation of RV function [3]. Tricuspid annular plane systolic excursion (TAPSE), RV fractional area change (RV FAC), tissue Doppler-derived tricuspid lateral annular systolic velocity (RV Sʹ), and three-dimensional RV ejection fraction (3D RV EF) are the commonly used parameters for assessing RV systolic function [3, 4]. However, these parameters are load dependent. As a reliable and superior indicator, RV global longitudinal strain (RV GLS) remains a load-related parameter because of the low ventricular elastance and the thin wall of the right ventricle [5, 6].

Recently, noninvasive RV myocardial work (RVMW) by echocardiography was demonstrated as a novel and reliable indicator to assess RV systolic performance [7, 8]. RVMW integrates RV GLS, pulmonary artery pressure, and cardiac cycle events, which provide more precise information than conventional RV systolic function parameters.

To date, noninvasive RVMW has not been applied to assess RV systolic function in patients with ASD. Therefore, the present study was designed to achieve the following objectives: (i) compare the RVMW between ASD patients and healthy controls; (ii) explore the correlations between the noninvasive RVMW and RV stroke volume (SV) and SV index measured by right heart catheterization (RHC) in ASD patients; and (iii) explore the possibility of using RVMW indices to evaluate myocardial performance among patients with ASD.

## Methods

### Study cohort

**Fig. 1:**
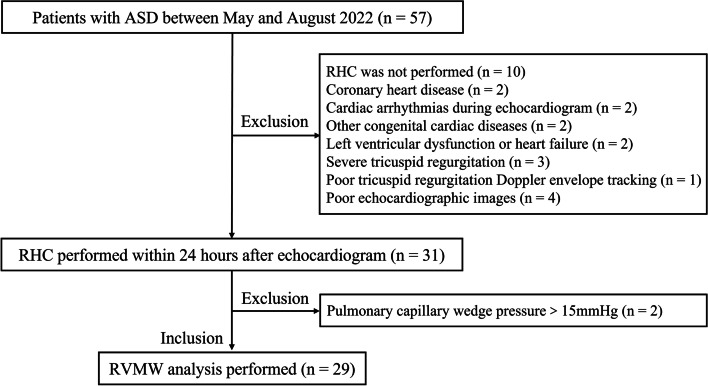
Study flow. ASD, atrial septal defect; RHC, right heart catheterization; RVMW, right ventricular myocardial work

A total of 57 ASD patients (> 17 years of age) were prospectively recruited in Xiamen Cardiovascular Hospital between May and August of 2022. The study flow chart is shown in Fig. 1. The exclusion criteria were as follows: RHC not performed within 24 h after echocardiogram, coronary heart disease, cardiac arrhythmias during the echocardiogram, other congenital cardiac diseases, left ventricular (LV) dysfunction or heart failure, severe tricuspid regurgitation (TR) [9], TR Doppler envelope of poor quality, poor echocardiography images, and pulmonary capillary wedge pressure > 15 mmHg [10]. After exclusion, 29 patients were finally included. An additional 29 age- and sex-matched subjects without cardiovascular diseases were enrolled as the control group. The Ethics Committee approved the study, and informed consent forms were obtained.

### Echocardiographic acquisition

Transthoracic echocardiographic images were obtained by a Vivid E95 ultrasound system (GE Vingmed Ultrasound) according to the recommended protocols [11, 12]. Two-dimensional and three-dimensional (3D) echocardiographic images were obtained by M5S and 4 V transducers, respectively. All echocardiographic images were stored over 3–4 consecutive cardiac cycles with the electrocardiogram connected. Datasets were analysed offline using EchoPAC (version 204).

### Echocardiographic measurements

**Fig. 2:**
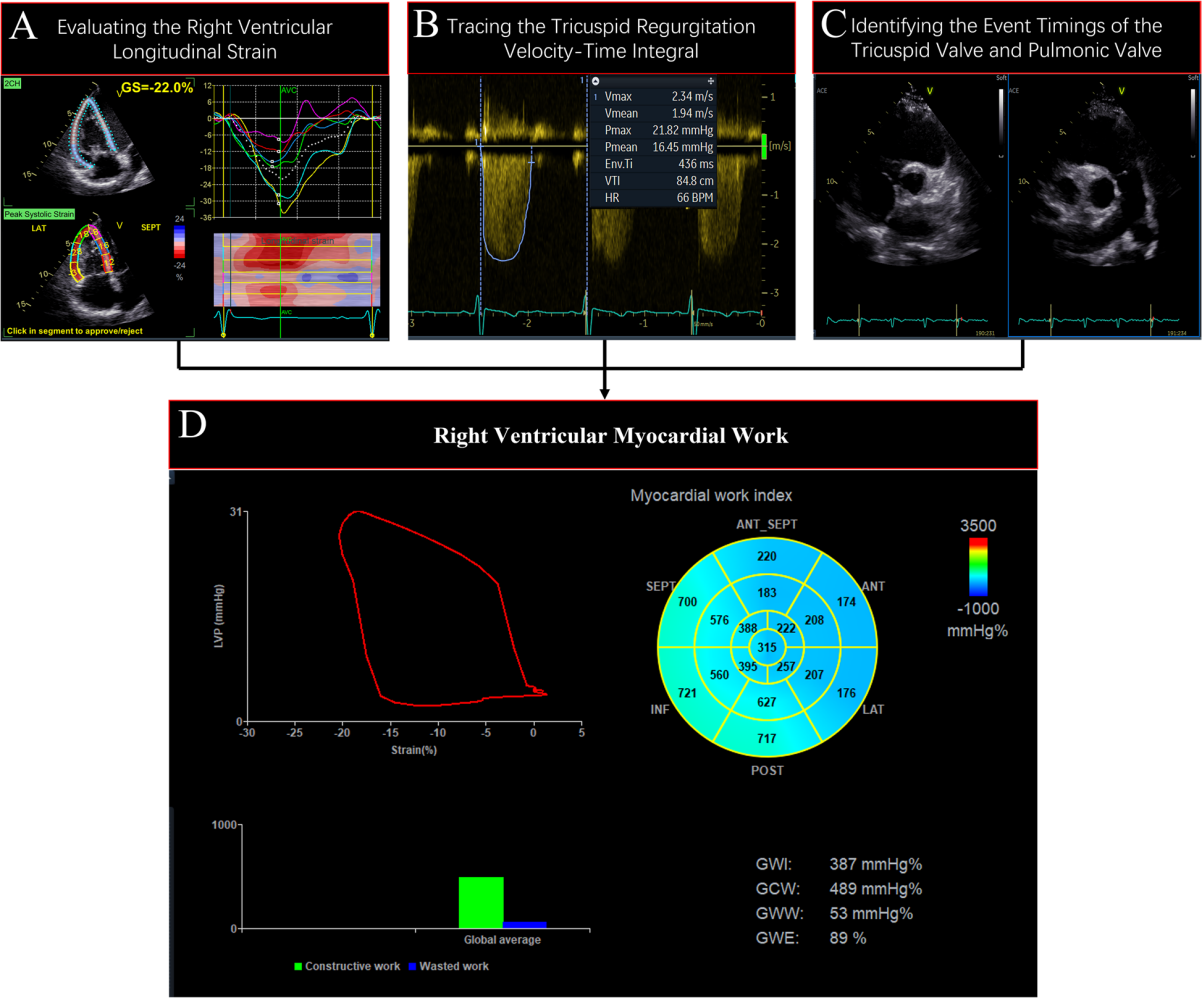
Process for calculating right ventricular myocardial work. **A** Evaluating the right ventricular longitudinal strain. **B** Tracking the TR velocity-time integral to assess the mean gradient pressure between the right ventricle and atrium. **C** Identifying the event timing of the tricuspid valve and pulmonary valve. **D** Obtaining right ventricular myocardial work by the right ventricular pressure-strain loop

The LV ejection fraction, TAPSE, RV FAC, RV Sʹ, RV basal diameter, and tricuspid annular diameter were measured in line with the current guidelines [3, 4, 13]. The 3D RV volume and RA volume were obtained by the software packages 4D Auto RVQ and 4D Auto LAQ, respectively. RV GLS and RV free wall longitudinal strain (RV FWLS) were assessed by tracing the endocardial border of the interventricular septum and the RV free wall (Fig. 2A) [12].

Systolic pulmonary artery pressure (SPAP) was estimated as follows: SPAP = 4 × (TR peak velocity)^2^ + RA pressure (estimated by the inferior vena cava) [3, 14]. The mean RV-RA gradient pressure was obtained by tracking the TR velocity-time integral (Fig. 2B) [15]. Mean pulmonary artery pressure (MPAP) equals the RA pressure plus the mean RV-RA gradient pressure. Diastolic pulmonary artery pressure (DPAP) was computed as follows: DPAP = 1.5 × MPAP − 0.5 × SPAP [3]. RVMW was analysed using the LV myocardial work (LVMW) assessment software package (AFI). Prognostic validation of LVMW was performed in several studies [16–18]. The event timings of tricuspid and pulmonic valves were obtained from visualization in the short-axis parasternal views (Fig. 2C). Then, RV GLS, SPAP, and DPAP were synchronized by valve event timings to create a noninvasive RV pressure–strain loop (RV PSL) (Fig. 2A-D). RVMW was calculated by integrating the product of the instantaneous RV pressure over time and the rate of segmental shortening to obtain myocardial work as a function of time during the cardiac cycle.

Four RVMW indices were obtained as follows:

(i) RV global work index (RVGWI): total work, the area of RV PSL from tricuspid valve closure to opening.
(ii) RV global constructive work (RVGCW): positive work, myocardial lengthening during isovolumic relaxation and shortening during systole.
(iii) RV global wasted work (RVGWW): negative work, myocardial shortening during isovolumic relaxation and lengthening during systole.
(iv) RV global work efficiency (RVGWE): the ratio of RVGCW to the sum of RVGCW and RVGWW.

### RHC

RHC was performed by experienced interventional cardiologists. A 6 F Swan Ganz catheter was inserted through the femoral or internal jugular vein under fluoroscopic guidance. RV systolic and diastolic pressure, pulmonary artery pressure, and pulmonary capillary wedge pressure were acquired at end-expiration. The ratio of pulmonary to systemic blood flow and LV and RV cardiac output were obtained by the Fick formula. RV SV was calculated as RV cardiac output divided by heart rate. The RV SV index and LV and RV cardiac indices were calculated as RV SV and LV and RV cardiac output divided by body surface area, respectively.

### Statistics

Categorical variables were expressed as numbers (percentage). The normality of continuous variables was verified by the Kolmogorov–Smirnov test. Based on the normality of the data, continuous variables were expressed as the mean (SD) or median (interquartile range), appropriately. Differences between the ASD and control groups were compared by the χ^2^ test, Student’s *t* test, and Mann‒Whitney *U* test as appropriate. Relationships between parameters of RV systolic function and invasively derived SV and SV index were investigated by Pearson or Spearman correlation as appropriate. Receiver operating characteristic (ROC) curves were analysed to determine optimal cutoff values to predict ASD and to calculate the area under the ROC curve (AUC), sensitivity, and specificity. Fifteen random subjects with ASD were selected for the calculation of intra-observer and inter-observer variabilities by Bland–Altman analysis and intraclass correlation coefficients. All data were processed using SPSS (version 26.0). A two-sided *P* value < 0.05 was considered indicative of statistical significance.

## Results

### Participant characteristics

Fifty-seven patients with ASD were enrolled in this study (Fig. 1). Forty-seven patients underwent RHC for clinical indications. Twenty-nine patients were included in the final analysis, and the rest of the patients were excluded based on the exclusion criteria. An additional 29 subjects without cardiovascular and pulmonary disease were set as the control group. The clinical characteristics are summarized in Table 1.

**Table 1:** Clinical characteristics of ASD patients and normal controls

Variables	ASD (*n* = 29)	Control (*n* = 29)	*P-*value
Male, *n* (%)	6 (21%)	6 (21%)	1.000
Age (years)	49.0 (32.0–58.0)	49.0 (34.5–51.0)	0.688
Height (cm)	160.4 ± 7.4	161.6 ± 6.1	0.488
Weight (kg)	57.0 (53.5–62.5)	58.0 (52.5–62.0)	0.651
BMI (m/kg^2^)	23.1 (20.9–25.1)	22.0 (20.1–23.6)	0.240
BSA (m^2^)	1.56 (1.53–1.68)	1.61 (1.52–1.67)	0.779
SBP (mmHg)	125 (116–142)	126 (107–133)	0.259
DBP (mmHg)	78 ± 11	74 ± 10	0.134
Hypertension, *n* (%)	6 (21%)		
Hyperlipidemia, *n* (%)	11 (42%)		
Diabetes, *n* (%)	4 (15%)		
NYHA Class III or IV, *n* (%)	5 (19%)		
NT-proBNP (pg/mL)	60.3 (34.8–128.0)		

Data are presented as mean ± SD or median (interquartile range) or as number (percentage). *ASD* Atrial septal defect, *BMI* Body mass index, *BSA* Body surface area, *DBP* Diastolic blood pressure, *NT-proBNP* N-terminal pro-B-type natriuretic peptide, *NYHA* New York Heart Association, *SBP* Systolic blood pressure

### Echocardiographic data

All enrolled patients had left-to-right colour Doppler shunts, and the median ASD size was 11.0 (8.0-18.5) mm. Table 2 summarizes the echocardiographic parameters of patients with ASD and controls. TAPSE, RV Sʹ, 3D RV end-diastolic volume, 3D RV end-systolic volume, 3D RV SV, RV basal diameter, tricuspid annular diameter, 3D RA volume, pulmonary artery pressure, and RV GLS were higher in the ASD group. LVEF, RV FAC, 3D RV EF, and RV FWLS were not significantly different between the two groups. Patients with ASD had a higher RVGWI, RVGCW, and RVGWW than the controls (RVGWI: 571.7 ± 203.2 mmHg% vs. 311.2 ± 98.7 mmHg%, *P < *0.001; RVGCW: 690.8 ± 254.8 mmHg% vs. 361.5 ± 101.3 mmHg%, *P < *0.001; RVGWW: 56.0 [37.5–75.0] mmHg% vs. 22.0 [9.0-37.5] mmHg%, *P < *0.001), while RVGWE showed no significant difference between ASD patients and controls.

**Table 2:** Comparison of echocardiographic parameters between ASD patients and normal controls

Variables	ASD (*n* = 29)	Control (*n* = 29)	*P*-value
LVEF (%)	63.6 ± 3.4	64.7 ± 2.9	0.173
TAPSE (mm)	25.4 ± 3.6	20.2 ± 3.6	<0.001
RV FAC (%)	46.6 ± 3.7	47.1 ± 4.4	0.689
RV Sʹ (cm/s)	15.0 ± 3.0	13.1 ± 1.8	0.004
3D RV end-diastolic volume (mL)	153 (117–193)	91 (79–98)	<0.001
3D RV end-systolic volume (mL)	67 (44–80)	37 (34–40)	<0.001
3D RV stroke volume (mL)	89 ± 25	53 ± 7	<0.001
3D RV EF (%)	57.6 ± 3.3	58.6 ± 1.9	0.140
RV basal diameter (mm)	44.7 ± 7.8	34.4 ± 3.7	<0.001
TA diameter (mm)	36.8 ± 6.2	26.9 ± 3.7	<0.001
3D RA maximum volume (mL)	50 (43–64)	36 (34–39)	<0.001
SPAP (mmHg)	35.4 (29.1–48.4)	21.9 (18.6–25.6)	<0.001
DPAP (mmHg)	21.1 (18.0-27.3)	16.3 (13.6–18.6)	<0.001
MPAP (mmHg)	25.9 (22.2–34.2)	13.6 (11.2–15.7)	<0.001
RV FWLS (%)	-25.2 ± 3.8	-24.4 ± 3.5	0.401
RV GLS (%)	-22.3 ± 3.0	-20.7 ± 2.6	0.030
RVGWI (mmHg%)	571.7 ± 203.2	311.2 ± 98.7	<0.001
RVGCW (mmHg%)	690.8 ± 254.8	361.5 ± 101.3	<0.001
RVGWW (mmHg%)	56.0 (37.5–75.0)	22.0 (9.0-37.5)	<0.001
RVGWE (%)	91.0 (89.0–93.0)	92.0 (89.0–97.0)	0.214

Data are presented as mean ± SD or median (interquartile range). *3D* Three-dimensional, *ASD* Atrial septal defect, *DPAP* Diastolic pulmonary artery pressure, *EF* Ejection fraction, *FAC* Fractional area change, *FWLS* Free wall longitudinal strain, *GLS* Global longitudinal strain, *LVEF* Left ventricular ejection fraction, *MPAP* Mean pulmonary arterial pressure, *RA* Right atrial, *RV* Right ventricular, *RVGCW* RV global constructive work, *RVGWE* RV global work efficiency, *RVGWI* RV global work index, *RVGWW* RV global work waste, Sʹ, tissue Doppler-derived tricuspid lateral annular systolic velocity, *SPAP* Systolic pulmonary artery pressure, *TA* Tricuspid annular, *TAPSE* TA plane systolic excursion

### RHC characteristics

RHC data of patients with ASD are summarized in Table 3. RV SV (114.7 ± 37.9 mL), RV SV index (71.2 ± 24.0 mL/m^2^), RV cardiac output (8.9 ± 3.7 L/min), RV cardiac index (5.5 ± 2.4 L/min/m^2^) were increased in patients with ASD. SPAP calculated by RHC showed no significant difference from SPAP estimated by echocardiography (38.0 [29.0-48.5] mmHg vs. 35.4 [29.1–48.4] mmHg, *P* = 0.320). The mean Qp/Qs ratio was 1.9 ± 0.8 in the patients with ASD.

**Table 3:** RHC characteristics of ASD patients

Variables	*n* = 29
RHC-derived SPAP (mmHg)	38.0 (29.0-48.5)
RHC-derived DPAP (mmHg)	7.0 (6.0-13.5)
RHC-derived MPAP (mmHg)	19.0 (15.0–25.0)
RV stroke volume (mL)	114.7 ± 37.9
RV stroke volume index (mL/m^2^)	71.2 ± 24.0
RV cardiac output (L/min)	8.9 ± 3.7
RV cardiac index (L/min/m^2^)	5.5 ± 2.4
LV cardiac output (L/min)	4.9 ± 1.6
LV cardiac index (L/min/m^2^)	3.0 ± 1.0
PCWP (mmHg)	8.6 ± 2.9
Qp/Qs	1.9 ± 0.8

Data are presented as mean ± SD or median (interquartile range). *ASD* Atrial septal defect, *DPAP* Diastolic pulmonary artery pressure, *LV* Left ventricular, *MPAP* Mean pulmonary artery pressure, *PCWP* Pulmonary capillary wedge pressure, *Qp/Qs* Ratio of pulmonary to systemic blood flow, *RHC* Right heart catheterization, *RV* Right ventricular, *SPAP* Systolic pulmonary artery pressure

### Relationship between parameters of RV systolic function and RHC parameters

The correlations between RHC-derived SV and SV index and the echocardiographic parameters of RV systolic function were calculated in the ASD group (Table 4 and Supplementary Fig. 1). Except for RV GLS, which was significantly correlated with RV SV and RV SV index (*r* = -0.478, *P* = 0.009 and *r* = -0.488, *P* = 0.007, respectively), none of the standard echocardiographic parameters of RV systolic function were significantly correlated with RV SV or the RV SV index. However, RVGWI showed moderate correlations with RV SV and SV index (*r* = 0.503, *P* = 0.005 and *r* = 0.521, *P* = 0.004, respectively), RVGCW showed weak correlations with RV SV and SV index (*r* = 0.440, *P* = 0.017 and *r* = 0.461, *P* = 0.012, respectively), and RVGWW showed weak correlations with RV SV and SV index (*r* = 0.444, *P* = 0.016 and *r* = 0.410, *P* = 0.027, respectively).

**Table 4:** Correlations between echocardiographic parameters of RV systolic function and invasive stroke volume and stroke volume index

*r*	RHC stroke volume	*P*-value	RHC stroke volume index	*P*-value
TAPSE	0.321	0.089	0.226	0.238
RV FAC	0.326	0.084	0.290	0.127
RV S’	0.360	0.055	0.319	0.092
3D RV EF	0.032	0.870	0.002	0.991
RV FWLS	-0.313	0.098	-0.339	0.072
RV GLS	-0.478	0.009	-0.488	0.007
RVGWI	0.503	0.005	0.521	0.004
RVGCW	0.440	0.017	0.461	0.012
RVGWW	0.444	0.016	0.410	0.027
RVGWE	0.106	0.586	0.114	0.557

*3D* Three-dimensional, *EF* Ejection fraction, *FAC* Fractional area change, *FWLS* Free wall longitudinal strain, *GLS* Global longitudinal strain, *RHC* Right heart catheterization, *RV* Right ventricular, *RVGCW* RV global constructive work, *RVGWE* RV global work efficiency, *RVGWI* RV global work index, *RVGWW* RV global work waste, *S* *ʹ* Tissue Doppler-derived tricuspid lateral annular systolic velocity, *TAPSE* Tricuspid annular plane systolic excursion

### ROC analysis

**Fig. 3:**
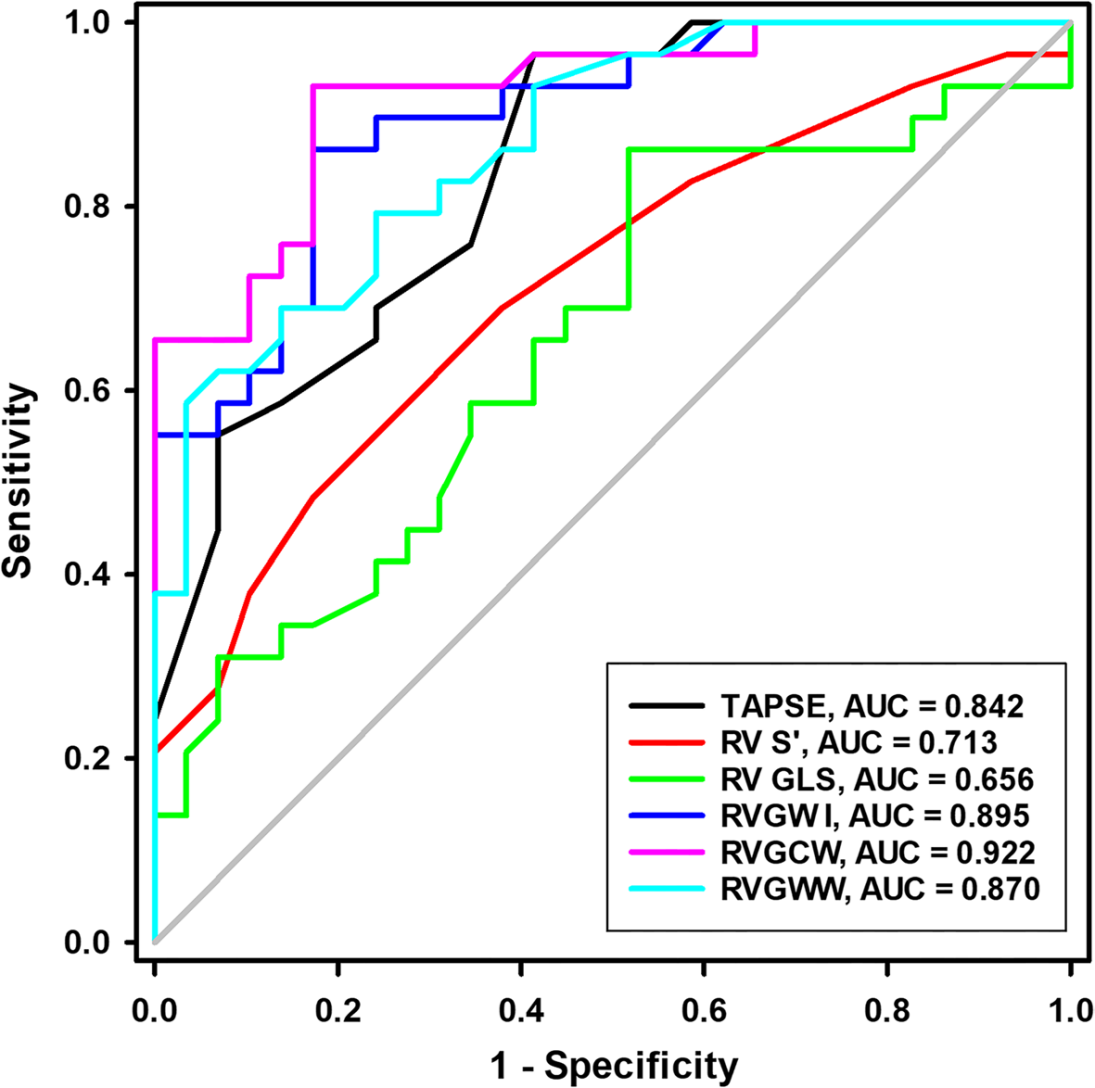
Receiver operating characteristic analysis of TAPSE, RV Sʹ, RV GLS, RVGWI, RVGWW, and RVGWE for predicting atrial septal defect. GLS, global longitudinal strain; RV, right ventricular; RVGCW, RV global constructive work; RVGWE, RV global work efficiency; RVGWI, RV global work index; RVGWW, RV global work waste; RV Sʹ, tissue Doppler-derived tricuspid lateral annular systolic velocity; TAPSE, tricuspid annular plane systolic excursion

ROC analysis was performed to determine whether standard echocardiographic parameters and RVMW indices could identify patients with ASD (Table 5; Fig. 3). The ROC analysis revealed that the optimal TAPSE, RV Sʹ, RV GLS, RVGCW, RVGWI, and RVGWW cutoff points were 20.2 mm (AUC = 0.842), 13.5 cm/s (AUC = 0.713), -19.8% (AUC = 0.656), 376.5 mmHg% (AUC = 0.895), 430.0 mmHg% (AUC = 0.922), and 45.5 mmHg% (AUC = 0.870), respectively.

**Table 5:** ROC analysis of echocardiographic parameters to identify atrial septal defect

Variables	AUC (SE)	*P*-value	AUC (95% CI)	Cutoff value	Sensitivity	Specificity
TAPSE (mm)	0.842 (0.050)	<0.001	0.744–0.940	20.2	96.6%	58.6%
RV-FAC (%)	0.499 (0.080)	0.994	0.342–0.656	43.9	86.2%	37.9%
RV Sʹ (cm/s)	0.713 (0.068)	0.005	0.580–0.846	13.5	69.0%	62.1%
3D RV EF (%)	0.365 (0.077)	0.078	0.215–0.516	61.6	20.7%	96.6%
RV FWLS (%)	0.553 (0.077)	0.489	0.403–0.703	-25.4	48.3%	69.0%
RV GLS (%)	0.656 (0.073)	0.042	0.514–0.798	-19.8	86.2%	48.3%
RVGWI (mmHg%)	0.895 (0.040)	<0.001	0.816–0.974	376.5	86.2%	82.8%
RVGCW (mmHg%)	0.922 (0.034)	<0.001	0.855–0.989	430.0	93.1%	82.8%
RVGWW (mmHg%)	0.870 (0.045)	<0.001	0.782–0.957	45.5	69.0%	86.2%
RVGWE (%)	0.405 (0.077)	0.216	0.255–0.556	87.5	86.2%	17.2%

*3D* Three-dimensional, *AUC* The area under receiver operating characteristic curve, *CI* Confidence interval, *EF* Ejection fraction, *FAC* Fractional area change, *FWLS* Free wall longitudinal strain, *GLS* Global longitudinal strain. *ROC* Receiver operating characteristic, *RV* Right ventricular, *RVGCW* RV global constructive work, *RVGWE* RV global work efficiency, *RVGWI* RV global work index, *RVGWW* RV global work waste, *Sʹ* Tissue Doppler-derived tricuspid lateral annular systolic velocity, *SE* Standard error, *TAPSE* Tricuspid annular plane systolic excursion

### Intra-observer and inter-observer variabilities in RVMW indices

Intra-observer and inter-observer variabilities of RVGWI, RVGCW, RVGWW, and RVGWE are presented in Table 6 and Supplementary Fig. 2, showing good reproducibility.

**Table 6:** Intra- and inter-observer variability of non-invasive RV myocardial work indices

	Intra-observer variability (*n* = 15)			Inter-observer variability (*n* = 15)		
	Bias	95% CI	ICC	Bias	95% CI	ICC
RVGWI (mmHg%)	1.5	-54.0 to 57.1	0.971	-7.2	-51.8 to 37.4	0.982
RVGCW (mmHg%)	3.5	-44.7 to 51.7	0.986	-0.7	-41.9 to 40.6	0.990
RVGWW (mmHg%)	-0.1	-23.0 to 22.8	0.850	1.4	-20.4 to 23.2	0.849
RVGWE (%)	0.1	-2.5 to 2.7	0.849	-0.1	-2.9 to 2.6	0.830

*CI* Confidence interval, *ICC* Intraclass correlation coefficient, *RV* Right ventricular, *RVGCW* RV global constructive work, *RVGWE* RV global work efficiency, *RVGWI* RV global work index, *RVGWW* RV global work waste

## Discussion

This study is a proof-of-concept study to identify the feasibility of using noninvasive RVMW in ASD patients. Assessing RVMW may enhance the understanding of the pathophysiology of RV myocardial systolic function in ASD patients.

### Changes in RV systolic function parameters in ASD

In our study, RV FAC showed no significant difference between ASD patients and controls, which was consistent with previous research [19]. TAPSE and RV Sʹ were higher in ASD patients than in controls, which was in line with previous studies [20, 21].

In patients with ASD, there was an increased volume load on the right ventricle, which subsequently led to an enlargement of the RV cavity [22, 23]. However, the 3D RV EF in ASD patients was not significantly different from that in controls, which may be due to preserved RV contractility in RV volume overload for long periods [24]. In addition, RV FWLS showed no significant difference between ASD patients and controls, and this result was consistent with the study by Dragulescu et al. [21]. However, the RV GLS was worse in ASD patients than in controls, perhaps because RV dilatation by increased preload led to augmented wall tension of the interventricular septum.

The increased RV preload of ASD patients leads to an increase in the volume of blood in the pulmonary circulation and ultimately increases the afterload [25]. The RVGWI and RVGCW reflected positive myocardial performance and increase as the afterload increases. Moreover, the increase in RVGWW may be related to remodelling of the cardiomyocytes under prolonged load and myocardial dyssynchrony in a state of increased RV afterload [26]. However, there was no significant difference in RVGWE between ASD patients and controls. This demonstrates that RV global myocardial systolic performance could be well preserved under long-term capacity loads as well as pressure loads in ASD patients.

### Superiority of RVMW in evaluating RV systolic function

Compared with TAPSE, RV FAC, RV Sʹ, and RV longitudinal strain, RVMW integrates myocardial systolic function, RV pressure and cardiac cycle into the RV PSL. The function of the right ventricle is more susceptible to afterload than that of the left ventricle [6]. In addition, RV dyssynchrony has a substantial impact on RV function [27, 28]. Theoretically, comprehensive evaluations of RV systolic performance could be derived from the four RVMW indices.

Except for RV GLS, none of the standard echocardiographic parameters were significantly correlated with RHC-derived SV or SV index. Conversely, the RVGWI, RVGCW, and RVGWE showed positive correlations with RHC-derived SV and SV index. According to the ROC analysis, the RVGWI, RVGCW, and RVGWE could be considered good predictors of ASD and are superior to load-dependent RV GLS. Although the correlations between the three RVMW indices and RHC-derived RV SV and SV index are weak and moderate, RVMW is the best noninvasive method to evaluate RV systolic function in ASD patients compare to standard RV systolic indices.

### Clinical implications

Conventional echocardiographic RV systolic function parameters were used to examine RV myocardial contractile performance in ASD patients, but none of these parameters incorporated the effect of pre- or afterload on the right ventricle [19, 21, 29–31]. As RV afterload is reflected in RVMW, the latter could expand the echocardiographic assessment of RV function in patients with untreated ASD.

### Limitations

This study is a single-centre study, and the sample size of ASD patients included was small. Noninvasive RVMW was not validated by radionuclide ventriculography or cardiovascular magnetic resonance. Additionally, RVMW was acquired by using a single-provider platform specifically designed for measuring LVMW. The RV GLS was calculated by measuring the strains of the interventricular septum and RV free wall because of the irregular and complicated RV anatomy [32]. Therefore, the RVMW derived by RV PSL is not as accurate as the LVMW derived by the LV pressure-strain loop [16]. Moreover, noninvasive RVMW should be validated by invasive RV PSL in the future.

## Conclusions

RVGWI, RVGCW, and RVGWW are feasible indicators that assess RV systolic function and correlate with RHC-derived SV and SV index in patients with ASD. Noninvasive RVMW may predict RV systolic function and correlate with RHC-derived SV and SV index in patients with ASD, with possible prognostic implications. Further studies are required to verify the clinical role of noninvasive RVMW.

## Supplementary Information

**Additional file 1: Supplementary Figure 1.** Correlations of RV GLS, RVGWI, RVGCW, and RVGWWwith RHC-derived stroke volume and stroke volume index. GLS, globallongitudinal strain; RHC, right heart catheterization; RV, right ventricular;RVGCW, RV global constructive work; RVGWE, RV global work efficiency; RVGWI, RVglobal work index; RVGWW, RV global work waste. 

**Additional file 2: Supplementary Figure 2.** The Bland–Altman analysis for assessinginter-observer variability of right ventricular global work index (RVGWI),right ventricular global constructive work (RVGCW), right ventricular global wasted work (RVGWW), and right ventricular globalwork efficiency (RVGWE).  

## Data Availability

The data and material underlying this article will be shared on reasonable request to the corresponding authors.

## Abbreviations

ASD: Atrial septal defect
RV: Right ventricular
TAPSE: Tricuspid annular plane systolic excursion
RV FAC: RV fractional area change
RV Sʹ: tissue Doppler-derived tricuspid lateral annular systolic velocity
3D: Three-dimensional
RV EF: RV ejection fraction
RV GLS: RV longitudinal strain
RVMW: RV myocardial work
RHC: Right heart catheterization
LV: Left ventricular
TR: Tricuspid regurgitation
RA: Right atrial
SPAP: Systolic pulmonary artery pressure
MPAP: Mean pulmonary artery pressure
DPAP: Diastolic pulmonary artery pressure
LVMW: LV myocardial work
RV PSL: RV pressure–strain loop
RVGWI: RV global work index
RVGCW: RV global constructive work
RVGWW: RV global wasted work
RVGWE: RV global work efficiency
SV: Stroke volume
ROC: Receiver operating characteristic.
