# Supplementary figures and images for: Noninvasive right ventricular work in patients with atrial septal defects: a proof-of-concept study
Source: Cardiovasc Ultrasound. 2023 May 20;21:10. doi: 10.1186/s12947-023-00306-8 (PMC10199586; doi:10.1186/s12947-023-00306-8)

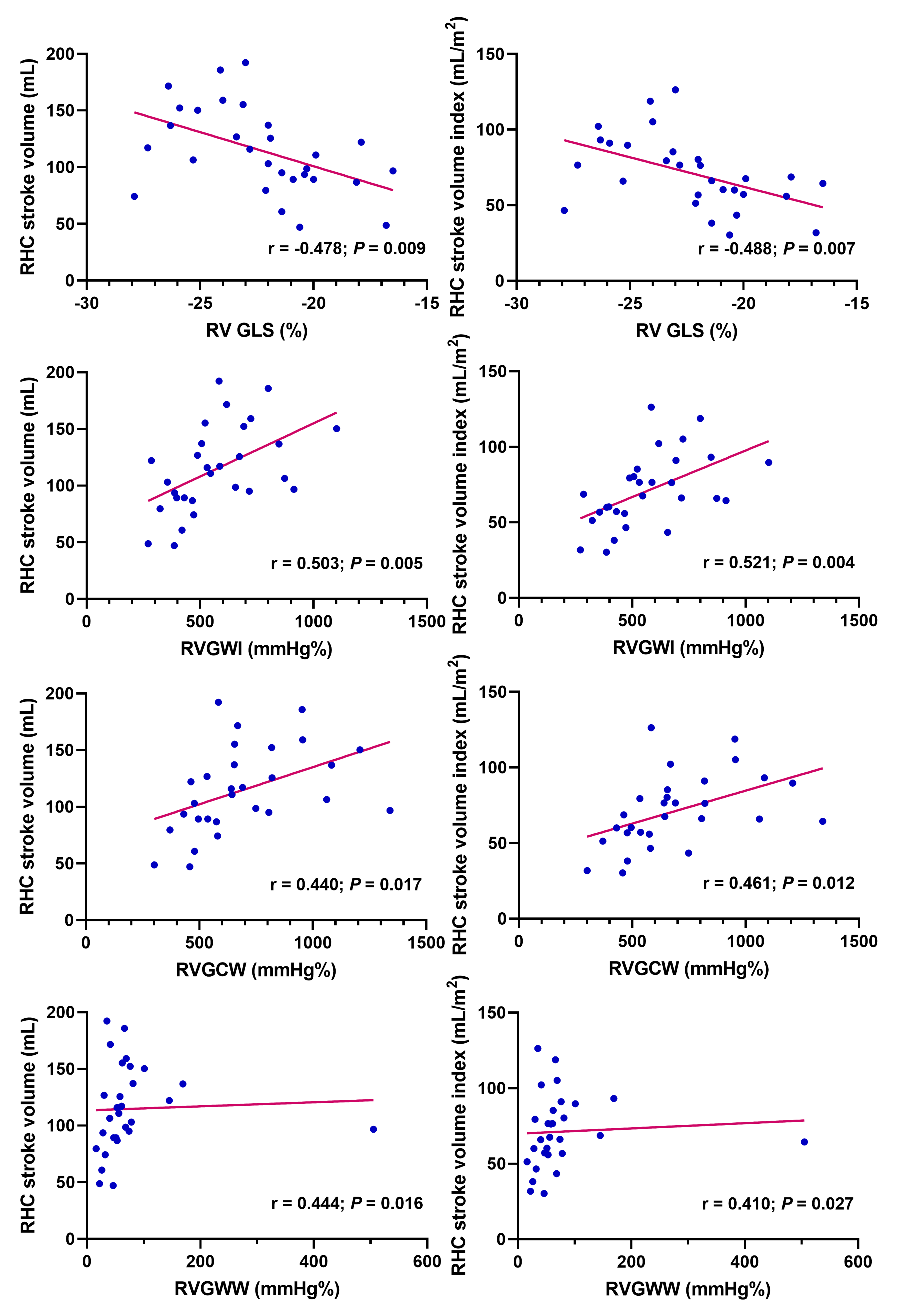

Supplement: Supplementary file 1 — Additional file 1: Supplementary Figure 1. Correlations of RV GLS, RVGWI, RVGCW, and RVGWWwith RHC-derived stroke volume and stroke volume index. GLS, globallongitudinal strain; RHC, right heart catheterization; RV, right ventricular;RVGCW, RV global constructive work; RVGWE, RV global work efficiency; RVGWI, RVglobal work index; RVGWW, RV global work waste. [file 12947_2023_306_MOESM1_ESM.tif]

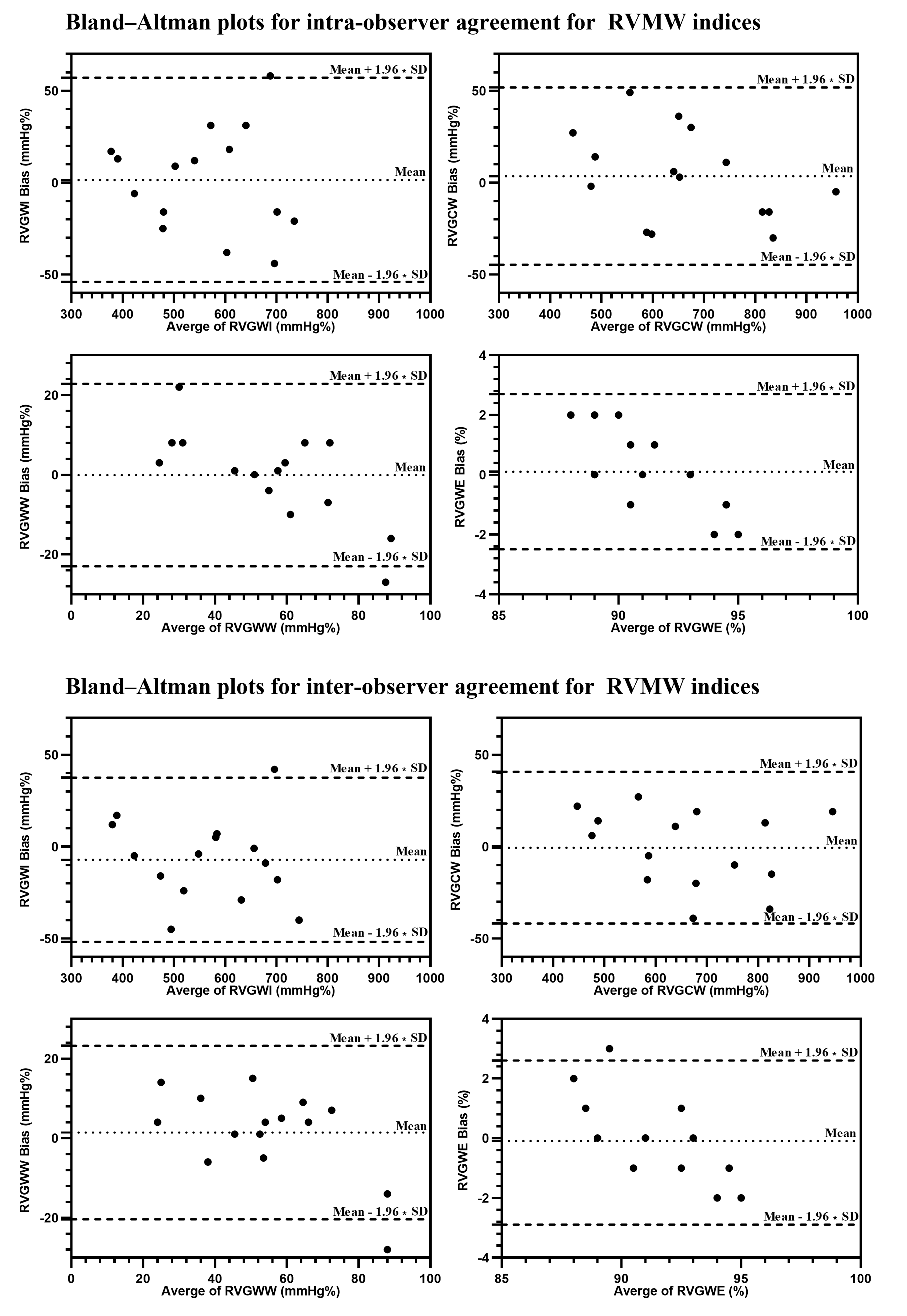

Supplement: Supplementary file 2 — Additional file 2: Supplementary Figure 2. The Bland–Altman analysis for assessinginter-observer variability of right ventricular global work index (RVGWI),right ventricular global constructive work (RVGCW), right ventricular global wasted work (RVGWW), and right ventricular globalwork efficiency (RVGWE). [file 12947_2023_306_MOESM2_ESM.tif]
